# Cataract-Causing S93R Mutant Destabilized Structural Conformation of βB1 Crystallin Linking With Aggregates Formation and Cellular Viability

**DOI:** 10.3389/fmolb.2022.844719

**Published:** 2022-03-14

**Authors:** Ling Ren, Lidan Hu, Ying Zhang, Jian Liu, Wanyue Xu, Wei Wu, Jingjie Xu, Xiangjun Chen, Ke Yao, Yibo Yu

**Affiliations:** ^1^ Eye Center of the Second Affiliated Hospital, Zhejiang University School of Medicine, Hangzhou, China; ^2^ National Clinical Research Center for Child Health, The Children’s Hospital, Zhejiang University School of Medicine, Hangzhou, China; ^3^ Institute of Translational Medicine, Zhejiang University School of Medicine, Hangzhou, China; ^4^ Eye Center of Zhejiang Hospital, Zhejiang University School of Medicine, Hangzhou, China

**Keywords:** βB1-crystallin, mutation, protein misfolding, aggregation, cellular apoptosis, environmental stress

## Abstract

Cataract, opacity of the eye lens, is the leading cause of visual impairment worldwide. The crucial pathogenic factors that cause cataract are misfolding and aggregation of crystallin protein. βB1‐crystallin, which is the most abundant water‐soluble protein in mammalian lens, is essential for lens transparency. A previous study identified the missense mutation βB1‐S93R being responsible for congenital cataract. However, the exact pathogenic mechanism causing cataract remains unclear. The S93 residue, which is located at the first Greek‐key motif of βB1‐crystallin, is highly conserved, and its substitution to Arginine severely impaired hydrogen bonds and structural conformation, which were evaluated *via* Molecular Dynamic Simulation. The βB1‐S93R was also found to be prone to aggregation in both human cell lines and *Escherichia coli*. Then, we isolated the βB1‐S93R variant from inclusion bodies by protein renaturation. The βB1-S93R mutation exposed more hydrophobic residues, and the looser structural mutation was prone to aggregation. Furthermore, the S93R mutation reduced the structural stability of βB1-crystallin when incubated at physiological temperature and made it more sensitive to environmental stress, such as UV irradiation or oxidative stress. We also constructed a βB1-S93R cellular model and discovered that βB1-S93R was more sensitive to environmental stress, causing not only aggregate formation but also cellular apoptosis and impaired cellular viability. All of the results indicated that lower solubility and structural stability, sensitivity to environmental stress, vulnerability to aggregation, and impaired cellular viability of βB1-S93R might be involved in cataract development.

## Introduction

Protein misfolding and aggregation are associated with many pathological disorders in humans, such as Neurodegenerative diseases, Cataract, and Type II diabetes (Dobson et al., 2001; Konarkowska et al., 2006; Andersen, 2006). Proteins spontaneously fold into specific and compact structures in order to carry out their functions efficiently. Protein might misfold and form insoluble aggregates under pathological conditions (Yang and Gruebele, 2003; Alam et al., 2017; Finkelstein, 2018). Cataract, which is characterized by opacity of the eye lens, is a prevalent disease associated with protein aggregation and the leading cause of blindness and visual impairment worldwide (Asbell et al., 2020). Misfolding and aggregation of different lens crystallins, which account for about 90% of the water soluble proteins of the lens, are considered the major pathogenic factors responsible for all types of cataracts (Luo et al., 2021). Furthermore, mutations in various genes have been linked to both age-related and congenital cataracts, and among the more than 100 genes reported in congenital cataracts, crystallin mutations account for almost half of the total number of mutations, although most of their pathogenetic mechanisms causing cataracts remain unclear (Li et al., 2020).

Lens crystallins are classified into two major families: **α**-crystallins and the **β/γ**-crystallin superfamily. **α**-crystallins are small heat shock proteins with molecular chaperones properties that prevent protein misfolding and aggregation in the lens, whereas **β/γ**-crystallins function as the main structural proteins (Li et al., 2020). The βB1-crystallin of the **β/γ**-crystallin superfamily is the most abundant and water-soluble protein in mammalian lenses. Because nascent proteins cannot be synthetized in lens fiber cells, the stability and solubility of βB1-crystallins at high concentrations over a lifetime is vital for the lens to maintain transparency and proper refractive index (Moreau and King, 2012), (Bassnett, 2009). Thus, human **β/γ**-crystallins have developed a strong ability to resist and avoid the damages caused by UV irradiation and oxidative stress, both of which have been proved to be crucial risk factors for cataracts (Giblin, 2000; Lou, 2003; Chen et al., 2006; Xu et al., 2009). Structurally, βB1-crystallins are characterized by two similar tertiary domains formed by two Greek-key motifs respectively, known as Greek-key motif 1–4 (Andley, 2007; van Montfort et al., 2009; Mishra et al., 2014). The Greek-key motif 4 is a hotspot for cataract research, but the Greek-key motif 1 receives relatively less attention, despite having many reported mutations (Cat-Map; http://cat-map.wustl.edu/).

In 2019, the S93R missense mutation of βB1-crystallin was identified in a four-generation Chinese family. All affected family members presented with bilateral and progressive cataracts from birth (Jin et al., 2020). S93 is structurally located in the first Greek-key motif, and the substitution to Arginine might affect not only the local structure around the mutation spot, but also the following three Greek-key motifs, eventually leading to significant changes in global structure. According to research, the S93R mutation disrupts the formation of two hydrogen bonds that aid in the stabilization of local structures, resulting in the formation of incorrect hydrogen bonds that damage both local and global structures (Jin et al., 2020). The present research aimed to study and compare the simulated structures and biophysical features of the wild type (WT) and the S93R mutant of βB1-crystallin. The results revealed that the S93R mutant significantly altered the protein structure and dramatically reduced the structural stability of βB1-crystallin. The S93R-βB1 was more prone to form aggregation, more sensitive to environmental stresses, and potentially toxic to human cells, which all contributed to the occurrence of congenital cataracts.

## Materials and Methods

### Materials

*Escherichia coli* Rosetta (DE3) cells were obtained from Tiangen Biotech. The HEK 293T and HLE-B3 cell lines were acquired from the American Type Culture Collection.

Isopropyl 1-thio-β-D-galactopyranoside (IPTG), Triton X-100, and NP40 solution were obtained from Sangon Biotech. The phenylmethylsulfonyl fluoride (PMSF) and 1-anilinonaphthalene-8-sulfonate (ANS) solutions were purchased from Sigma-Aldrich. Dulbecco’s modified Eagle’s medium (DMEM), DMEM/F-12 (1:1) medium, Lipofectamine 2000, Lipofectamine 3000, and Alexa Fluor Plus 555 antibodies were provided by Invitrogen. The GFP Tag Mouse antibody was procured from Proteintech. Propidium Iodide (PI) and Annexin V-Alexa Fluor 647 were obtained from Yeasen Biotech. All other reagents were local products with analytical grade.

### Plasmid Construction and Protein Purification

The human βB1–crystallin gene was cloned from a human lens cDNA library, and the S93R mutant of βB1 was constructed *via* site-directed mutagenesis. The WT and S93R genes were then inserted into the prokaryotic vector pET28a and the eukaryotic vector pEGFP-N1 for exogenous expression in *E. coli* Rosetta (DE3) cells and human cells, respectively, following previously described steps (Qi et al., 2016; Zhu et al., 2018). Briefly, the *E. coli* cells were first cultivated in Luria-Bertani medium at 37°C for 4 h and then treated with 0.01 mM isopropyl 1-thio-β-D-galactopyranoside (IPTG) at 16°C for 16 h to induce recombinant protein overexpression. Next, the target recombinant proteins were isolated from the supernatant of cell lysates using a Ni-NTA affinity column and further purified on the ÄKTA explorer through the HiLoad 16/600 Superdex 200 prep-grade column with 1 mM phenylmethylsulfonyl fluoride (PMSF). The purified proteins were dissolved in buffer A (20 mM Na_2_HPO_4_, 150 mM NaCl, and 1 mM EDTA, *pH* = 7.4) and concentrated before being stored at −80°C. The purity of the target proteins was verified using SDS-PAGE, which revealed that WT was more than 95% pure.

### Protein Refolding From the Inclusion Bodies

According to the SDS-PAGE analysis, the S93R proteins barely dissolved in the supernatant of cell lysates regardless of the cultivation temperatures and the concentrations of IPTG, indicating that the majority of the mutant proteins existed in inclusion bodies. Therefore, unlike the WT proteins, the S93R proteins were extracted from inclusion bodies in fully denatured status and then renatured using previously described methods (Qi et al., 2016). Briefly, the inclusion bodies were purified from the precipitation of cell lysates and then dissolved in buffer B (20 mmol/L Na_2_HPO_4_, 500 mmol/L NaCl and 6 mol/L GdnHCl, pH 7.0). The denatured S93R proteins were soluble and retrievable using Ni-NTA affinity and HiPrep 26/10 desalting columns in the same buffers as the WT proteins but with additional 6 mol/L GdnHCl. The purified proteins were concentrated to 1 ml and carefully diluted in buffer A at 0.1 ml/min until protein aggregation was observed. Next, the protein solution was incubated at 4°C for 16 h. The refolded S93R proteins were then found in the supernatant fraction after centrifugation. The refolded proteins were concentrated, and SDS-PAGE was used to ensure that the purity was greater than 95%.

### Spectroscopic Detection

The concentrated WT and the S93R proteins were diluted in buffer C (20 mM Na_2_HPO_4_, 150 mM NaCl, 0.8 mol/L GdnHCl and 1 mM EDTA, pH = 7.4) to the concentration of 10 μM for all samples measured using spectroscopy. Far-UV circular dichroism (CD) was measured using a Chirascan spectrophotometer. The path-length was 0.5 mm, and the range of scanning wavelength was from 200 to 250 nm. The intrinsic fluorescence and the extrinsic ANS fluorescence were measured using a F-4600 fluorescence spectrophotometer (Hitachi, Tokyo, Japan) using previously described methods and parameters (Yang et al., 2020; Fu et al., 2021; Liu et al., 2022). The excitation wavelength for intrinsic fluorescence was 280 nm for Trp and Tyr fluorescence and 295 nm for Trp fluorescence. The scanning wavelength of the intrinsic fluorescence ranged from 260 to 400 nm. For the temperature-gradient heating experiments, the protein samples were heated stepwise from 20 to 90°C at an interval of 2°C using the F-4600 fluorescence spectrophotometer. At each temperature, the samples were incubated for 2 min before Trp fluorescence was measured. The melting midpoint temperature (Tm) was calculated using the equation described previously (Zhu et al., 2018). Extrinsic ANS fluorescence was excited at 380 nm light, and the range of scanning wavelength was 400–700 nm. The 1-anilinonaphthalene-8-sulfonate (ANS) solution was added to protein samples at a 50:1 M ratio (ANS:protein) before measurement. A_400_ values was obtained by measuring the absorbance of protein samples at 400 nm light using an Evolution 300 Security UV/Vis Spectrophotometer (Thermo Fisher).

### Cell Model Establishment and Immunofluorescence

The HEK 293T cells were cultivated in DMEM with 10% FBS, whereas the HLE-B3 cells were cultured in DMEM/F-12 (1:1) with 20% FBS. Both cell lines were incubated in 5% CO_2_ at 37°C. The WT and S93R genes were exogenously overexpressed in both cell lines to establish the cataract cell models, and the protein distribution within the cells were visualized using immunofluorescence, as previously described (Xu et al., 2021). In brief, the WT and S93R-fused plasmids were transfected into the HEK 293T cells using Lipofectamine 2000 and the HLE-B3 cells were transfected using Lipofectamine 3000. After 5 h of transfection, the cells were cultured in fresh DMEM with 10% FBS or DMEM/F-12 (1:1) medium with 20% FBS at 37°C for 24 h, and fixed by 4% paraformaldehyde for 20 min. The cells were then washed three times with PBS buffer before being treated with 0.4% Triton X-100 for 10 min and blocked by 10% FBS for 50 min. The nuclei were identified using DAPI, the p62-detected aggregates were visualized using Alexa Fluor Plus 555 antibody, whereas the recombinant WT and S93R proteins were observed using the GFP tag. Immunofluorescence images were captured using a Leica DMi8 confocal microscope system, whereas a Fiji software was used to analyze the percentages of cells with aggregates and the area of protein aggregates in 10 random viewing fields that were from three independent experiments.

### Western Blot and SDS-PAGE Analyses

The HEK 293T cells were lysed in 1% NP40 solution after transfection and 24 h of cultivation. To perform western blot analysis, the total (T) protein solution was divided into a supernatant (S) fraction and a precipitated (P) fraction using high-speed centrifugation. The GFP-tagged WT and S93R proteins were detected using the GFP Tag Mouse antibody, and incubated at 4°C for 12 h before being treated with an anti-mouse secondary antibody. The Image Lab software (BioRad) was used to visualize and analyze the intensities of the protein bands. For SDS-PAGE, the WT and S93R proteins expressed in *E. coli* Rosetta cells were also divided into total (T), supernatant (S), and precipitated (P) samples. The gels were submerged in Coomassie blue dye for 30 min and rinsed with water to visualize the protein bands. The intensity of the protein bands was visualized using Image Lab software.

### Cell Viability and Apoptosis Assay

Cell viability of the transfected cells was determined using the Cell Counting Kit-8 (CCK-8) according to the manufacturer’s instructions. Briefly, the WT and S93R-fused plasmids were transfected into the HEK 293T cells seeded in 96-well culture plates. After 6, 24, 48, and 72 h of cultivation, 10 μl of the CCK-8 solution was added to each well. Then the cells were incubated at 37°C for 1 h, and the OD values were measured at wavelength 450 nm using a microplate reader (BioRad). For cell apoptosis assay, HEK 293T cells transfected with the WT and S93R genes were collected after 24 h of cultivation and stained with PI and Annexin V-Alexa Fluor 647. The percentages of apoptotic and necrotic cells were determined using flow cytometry on a CytoFLEX LX flow cytometer (Beckman Coulter).

### Molecular Dynamics Simulations

The structure file of βB1-crystallin (PDB code: 1OKI) was downloaded from the Protein Data Bank, and the S93R variant was constructed by Pymol, which is a molecular graphics system (http://www.pymol.org/). All simulations were performed using GROMACS (Version. 2018.5.1.2) under CHARMM36 force field. The molecular dynamics (MD) simulations were carried out in a cubic water box with 150 mM NaCl. The minimal distance from the edges of the cubic box to the protein surface was set to 0.6 nm. After energy minimization, the system was equilibrated for 5 ns under NVT and NPT conditions at 310 K. The simulations were run at 2 fs time steps for 100 ns. Then, the values of backbone root mean square deviation (RMSD), backbone root mean square fluctuation (RMSF), and solvent accessible surface area (SASA) were analyzed. Finally, the Visual Molecular Dynamics V.1.9.3 (Phillips et al., 2005) was used to process and analyze the simulated trajectories. All structural figures were generated with Pymol or Chimera software.

## Results

### The S93 is Highly Conserved and Probably Crucial to the Structural Stability of βB1–Crystallin

**FIGURE 1:**
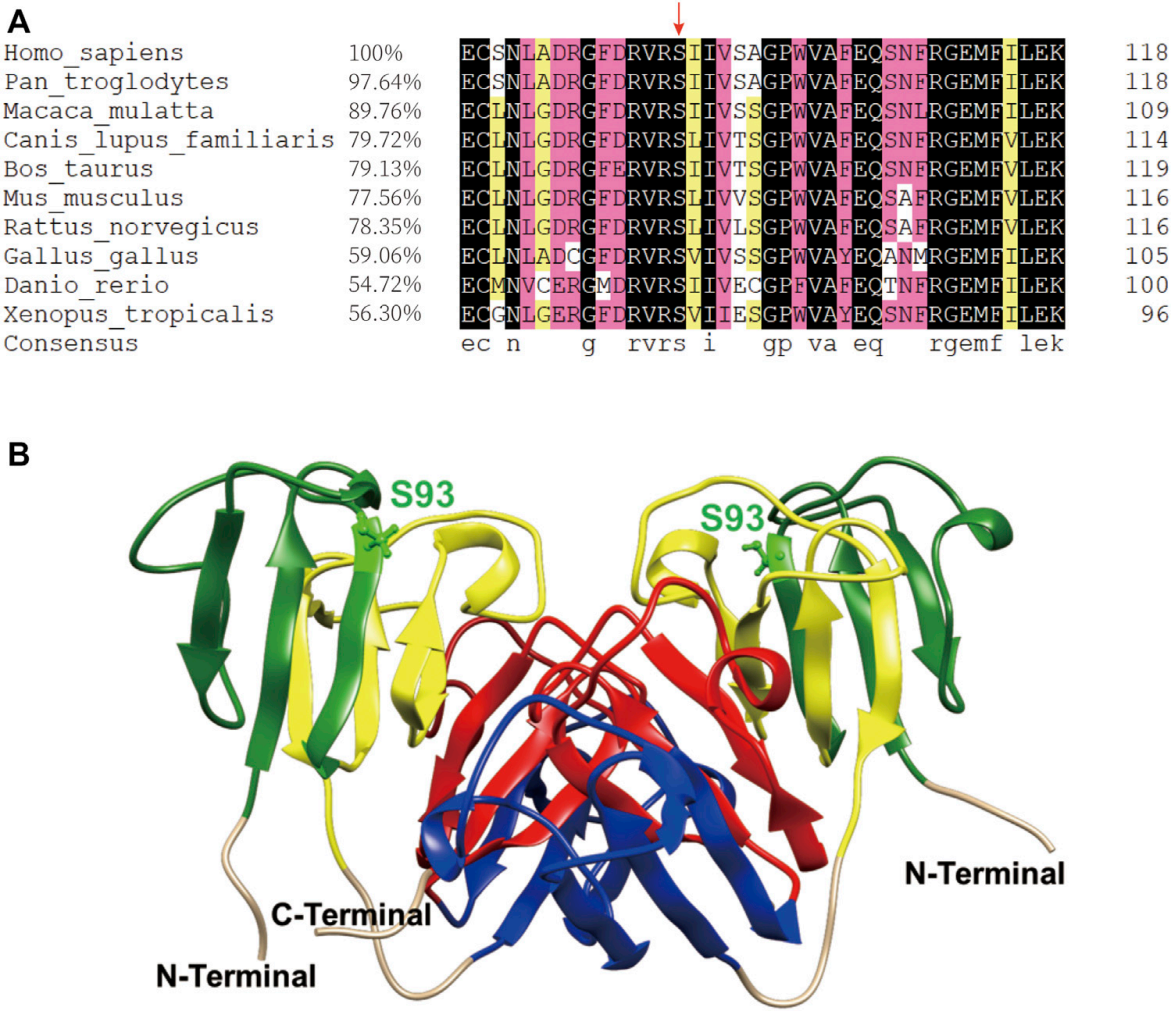
The S93 is highly conserved and probably crucial to the structural stability of βB1–crystallin. **(A)** Amino acid sequence alignment of βB1–crystallin in vertebrates. The 93rd serine residue is highly conserved as indicated by the red arrow. **(B)** The dimeric structure of the βB1-WT. The S93 is shown as balls and sticks. The first Greek-key motif to the fourth Greek-key motif is marked in green, yellow, blue and red, respectively.

The βB1-crystallin amino acid sequence alignment revealed that the 93rd serine residue remained highly conserved during the evolution of vertebrates (Figure 1A). Furthermore, previous studies of sequence alignment of the β/γ-crystallins reported that the S93 residue is conserved in the β/γ-crystallin superfamily in many species (Bloemendal et al., 2004; Jin et al., 2020). From the dimeric structure of the βB1-WT, S93 is located in the first Greek key motif and structurally close to the second Greek key motif (Figure 1B), implying that this site probably plays an important role in maintaining structural stability of βB1–crystallin.

### The S93R Mutation Promotes Protein Aggregation in Both Human Cell Lines and *E. Coli* Rosetta (DE3) Cells

**FIGURE 2:**
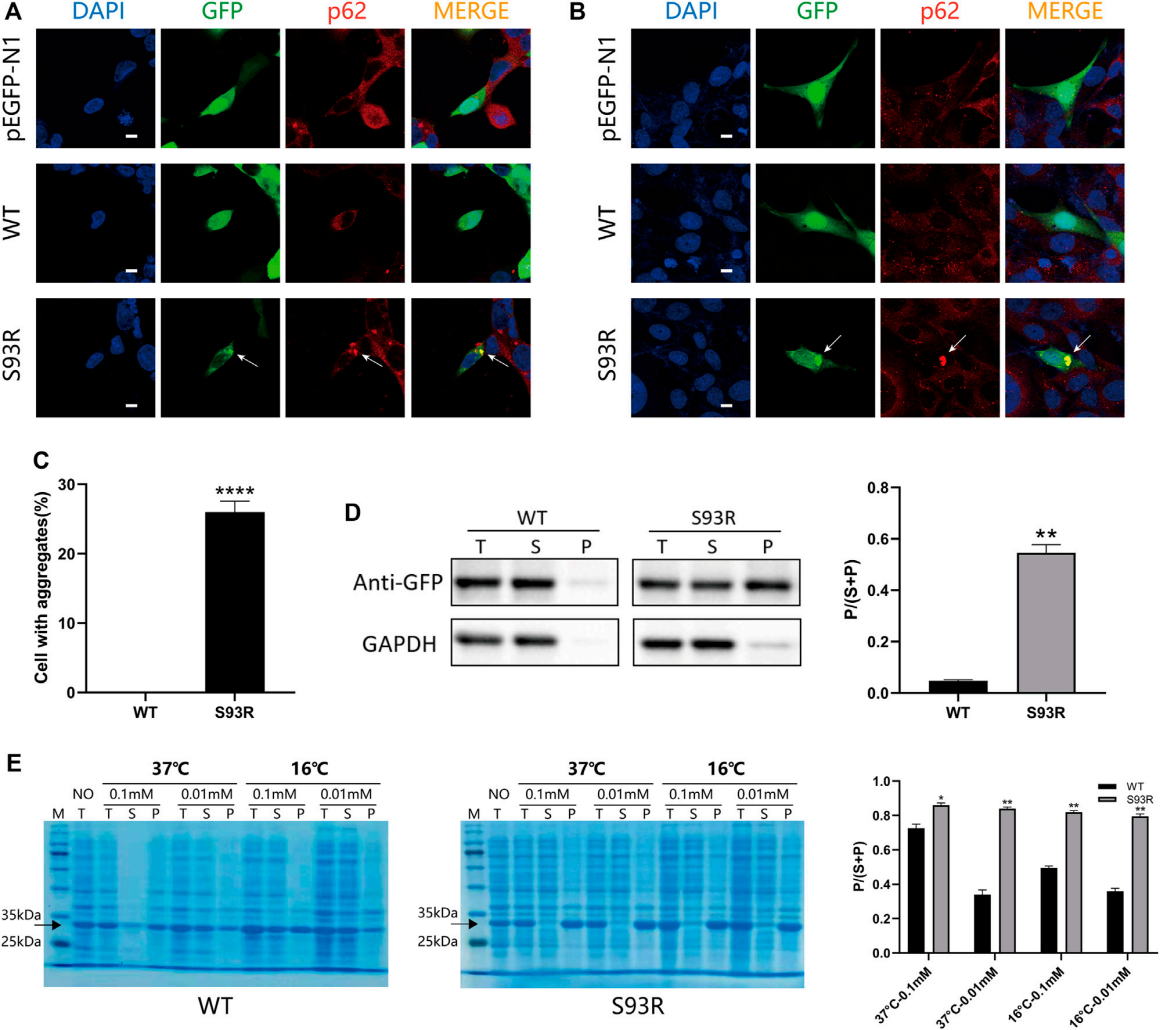
The S93R mutation promoted protein aggregation in both human cell lines and *E. coli Rosetta* (DE3). **(A)** Representative confocal images of HEK 293T cells overexpressing the EGFP-fused WT and S93R mutant proteins. An empty vector of pEGFP-N1 was transfected as a control group. The nucleus was stained with DAPI (blue). The overexpressed proteins were visualized using the GFP tag (green). Protein aggregates (indicated by white arrows) were recognized using the marker protein p62 (red). Scale bars: 10 μm. **(B)** Representative confocal images of HLE-B3 cells overexpressing the EGFP-fused WT and S93R mutant proteins. The white arrows indicate protein aggregates. Scale bars: 10 μm. **(C)** Quantitative analysis of protein aggregation in HEK 293T cells. The percentages of cells with aggregates were obtained by calculating the ratio of cells with intracellular protein aggregates which colocalized with p62 in 10 random viewing fields from three independent experiments. **(D)** Representative western blot results and the densitometric findings of WT and S93R protein overexpression in HEK 293T cells. Protein distribution is represented by the ratio of the densitometric values of P and that of S + P. T: total; S: supernatant; and P: precipitate. **(E)** Representative SDS-PAGE results and densitometric analysis of the distribution of WT and S93R proteins expressed in *E. coli* Rosetta cells when cultivated at different temperatures and IPTG concentrations, indicating that the mutated proteins were mainly present in inclusion bodies regardless of the culture conditions. The gels were stained with Coomassie blue. NO: no IPTG introduced. * means *p* < 0.05, ** means *p* < 0.01, and *** means *p* < 0.0001.

To observe the behaviors and distribution of the WT and S93R proteins in both human and prokaryotic cells, the WT and S93R genes were exogenously expressed in two kinds of human cell lines and *E. coli* Rosetta (DE3) cells (Figure 2). In the human cells, the GFP-fused WT protein dispersed uniformly in the cytosol and nucleus, which was consistent with the distribution pattern of the control group transfected with an empty pEGFP-N1 vector. However, the S93R mutant formed many intracellular protein aggregates that colocalized with p62, a classic aggresome marker protein (Figures 2A,B). A quantitative analysis of protein aggregation showed that the percentages of cells with protein aggregates differed significantly between the WT and S93R groups (Figure 2C). The proteins overexpressed in HEK 293T cells were then prepared for a western blot analysis. The western blot and the densitometric analyses showed that the WT protein mainly existed in the supernatant, while a considerable amount of S93R protein appeared in the precipitate (Figure 2D). For *E. coli* Rosetta (DE3) cells, the results of SDS-PAGE and densitometric analysis suggested that the amount of WT proteins in the supernatant fraction increased when the cultivation temperature or IPTG concentration decreased. The optimal culture condition for obtaining soluble WT proteins was at 16°C with 0.01 mM IPTG. However, the S93R mutated proteins were predominant in the precipitate fraction regardless of the culture conditions, implying that the mutated proteins mainly accumulated in inclusion bodies and could hardly be obtained by purification procedures of soluble proteins (Figure 2E).

### The Refolded S93R Proteins Were Partially Renatured and had an Aggregation Propensity

**FIGURE 3:**
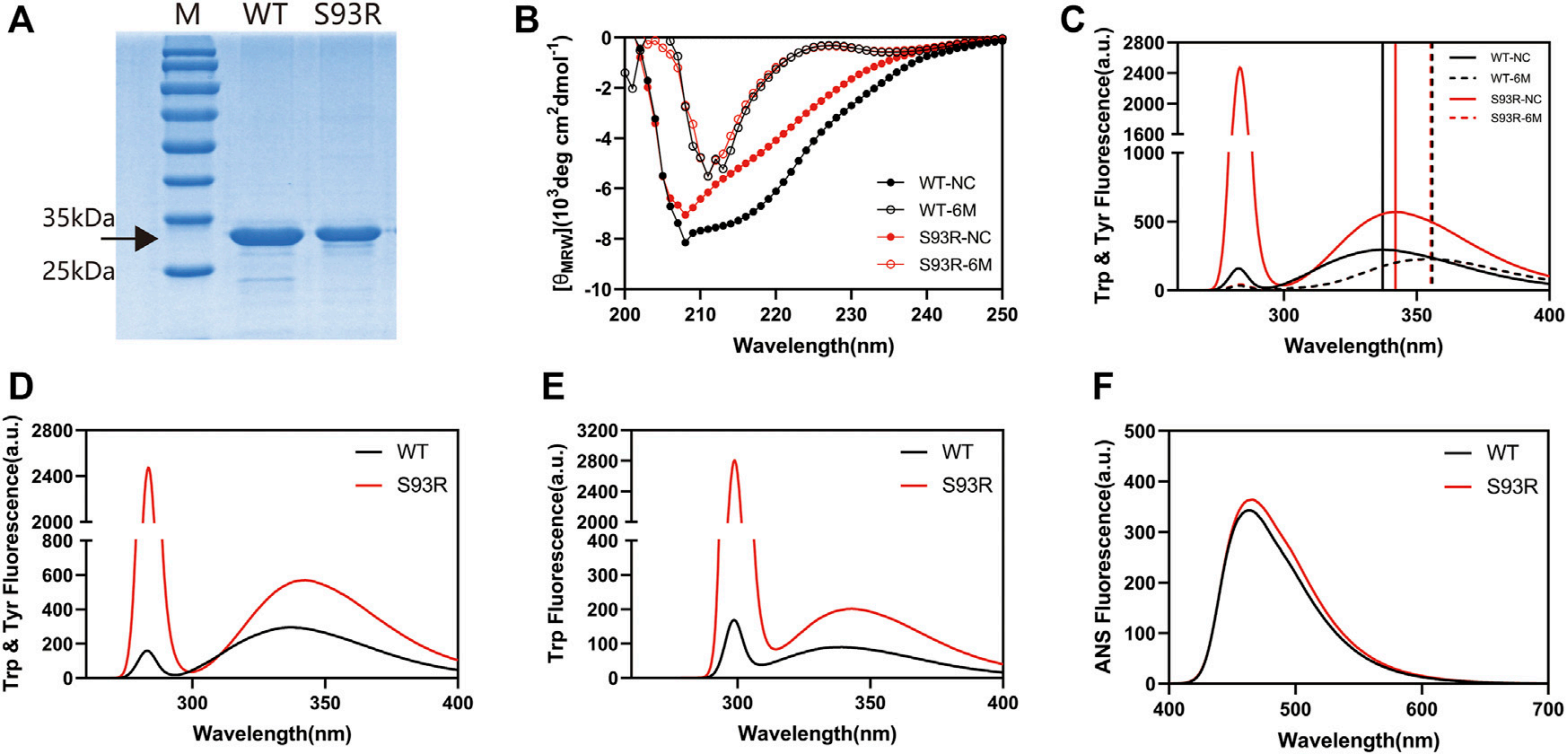
The basic biophysical properties of βB1-WT and refolded S93R mutant. **(A)** SDS-PAGE results of βB1-WT purified from the supernatant fraction of cell lysates and S93R mutant refolded from the inclusion bodies. The target protein bands are shown with a black arrow. The purity of both proteins was greater than 95%. **(B)** Far-UV CD spectra of βB1-WT and S93R mutant in 6 Mol/L GdnHCl treated groups. The values of CD spectra were converted into mean residue molar ellipticity [(θ_MRW_)]. NC denotes non-GdnHCl-treated control groups. **(C)** Intrinsic Trp and Tyr fluorescence spectra of βB1-WT and S93R mutant in 6 Mol/L GdnHCl treated groups. The excitation wavelength was 280 nm. **(D)** Intrinsic Trp and Tyr fluorescence spectra of the purified WT proteins and the refolded S93R mutant excited at 280 nm light. **(E)** Intrinsic Trp fluorescence spectra excited by 295 nm light. **(F)** Extrinsic ANS fluorescence spectra excited by 380 nm light.

The WT proteins were successfully purified from the supernatant fraction of *E. coli* cell lysates, however the S93R proteins were difficult to extract using the same procedures as the WT proteins. Therefore, the S93R proteins were completely denatured using 6 mol/L GdnHCl, purified from the inclusion bodies of *E. coli* cells and then renatured by dilution. To determine if the production protocols bias results, the obtained WT proteins were denatured and renatured following the same steps as the S93R proteins, and the intrinsic Trp fluorescence and far-UV CD showed that there were no major differences between the basic biophysical properties of WT proteins purified in native condition and in denaturant condition (Supplementary Figure S1). The SDS-PAGE analysis on the obtained protein samples revealed that the purities of both proteins were greater than 95% (Figure 3A). Furthermore, the obtained WT and S93R protein samples were compared to 6 mol/L GdnHCl treated groups using far-UV CD and intrinsic Trp & Tyr fluorescence. The results implied that the refolded S93R proteins were partially renatured as compared to the fully denatured status (Figures 3B,C). For intrinsic fluorescence, the E_max_ (maximum emission wavelength) of the refolded S93R proteins was comparable to that of the WT in both Trp & Tyr fluorescence and Trp fluorescence with a slight red-shift, as previously reported (Qi et al., 2016). However, S93R had significantly higher RLS (Rayleigh light scattering) intensity than the WT, suggesting that the refolded S93R formed small protein aggresomes and had a strong aggregation potential (Figures 3D,E). For extrinsic ANS fluorescence, S93R had a slight increase in ANS fluorescence in comparison to the WT, implying that the S93R proteins had higher hydrophobic residue exposure (Figure 3F).

### The S93R Mutation Reduced the Resistance of βB1–Crystallin to Environmental Stresses

**FIGURE 4:**
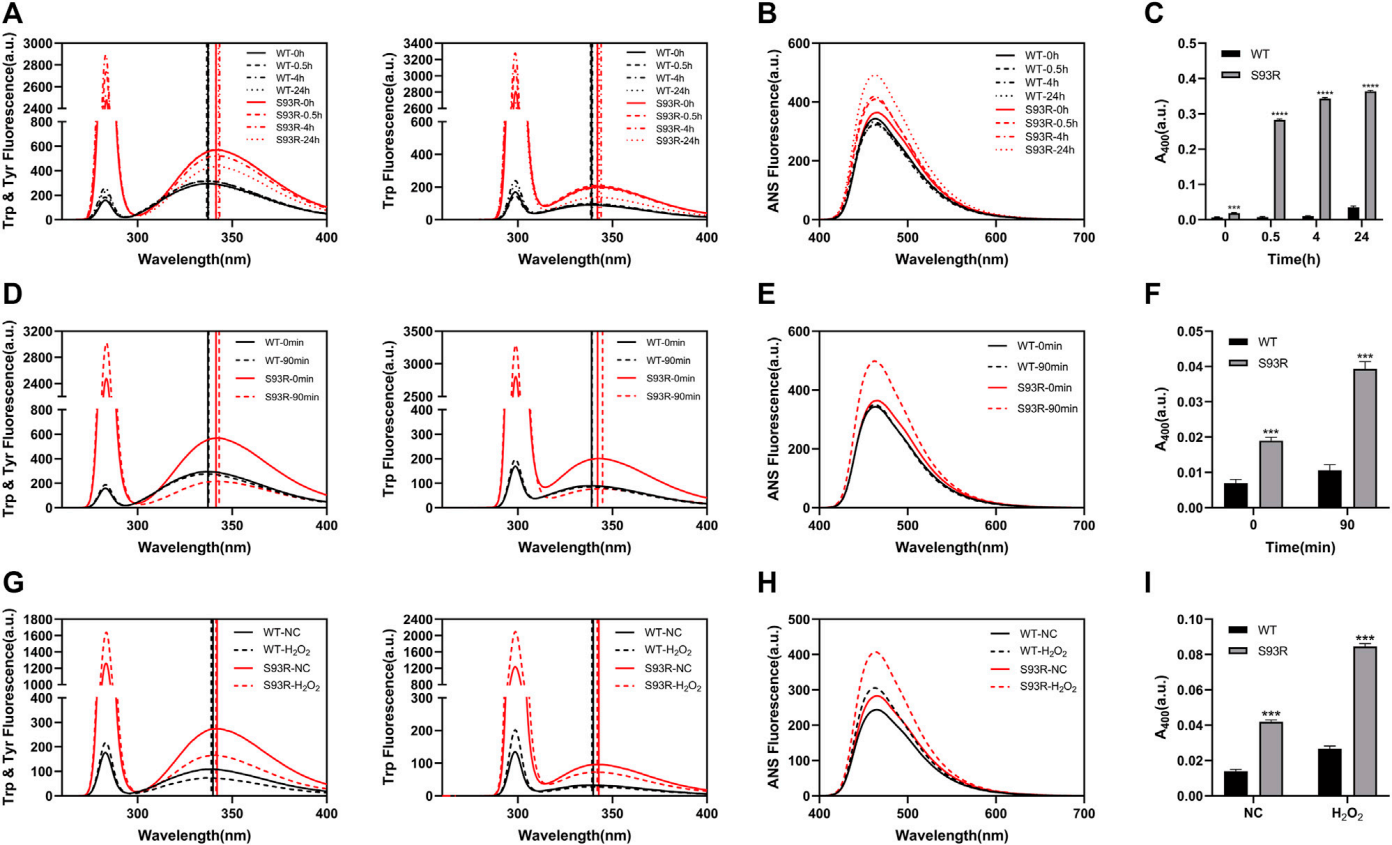
The S93R mutation reduced the resistance of βB1-crystallin to physiological temperature, UV irradiation, and oxidative stress. **(A–C)** Intrinsic fluorescence spectra, extrinsic ANS fluorescence spectra, and turbidity of the WT and refolded S93R protein solutions after incubation at 37°C. Turbidity is represented by the absorbance at 400 nm UV light (A_400_). **(D–F)** Intrinsic fluorescence spectra, extrinsic ANS fluorescence spectra, and turbidity of the WT and S93R proteins before and after 90 min of UV irradiation. **(G–I)** Intrinsic fluorescence spectra, extrinsic ANS fluorescence spectra, and turbidity of the WT and S93R proteins with and without oxidative stress. The H_2_O_2_-treated protein samples were incubated with 0.1 mM H_2_O_2_ at room temperature for 24 h. Protein samples without H_2_O_2_ treatment were also incubated at room temperature as a control group.

As indicated by previous work, protein stability and aggregation propensity are highly related (Booth et al., 1997). In this study, the results of temperature-gradient heating experiments showed that the WT remained in a native status as the temperature rose and started to denature at around 52°C, whereas the denaturation of S93R occurred at around 28°C. We also analyzed the T_m_ value of βB1-WT and S93R mutant, which was 60 and 32°C, respectively, (Supplementary Figure S2). Because the refolded S93R had an aggregation tendency, the protein samples were immediately subjected to different treatments and measurements after purification in order to assess the potential effects of S93R mutation on the βB1-crystallin (Figure 4). According to the intrinsic and extrinsic ANS fluorescence spectra, the WT had a slight increase on RLS intensity after incubation at 37°C, whereas the S93R mutant showed much higher RLS and ANS increase, indicating severe protein aggregation and hydrophobic residue exposure, as well as a slight red-shift of E_max_. (Figures 4A,B). In terms of turbidity, the results suggested that the WT was stable at physiological temperature. Nevertheless, the S93R aggregated rapidly after only 0.5 h of incubation at 37°C, and the turbidity increase steadily as the incubation duration prolonged, implying that the S93R was extremely unstable at physiological temperatures (Figure 4C). The fluorescence spectra results revealed that UV treatment had little effect on the WT, with just a slight increase of RLS intensity after 90 min of UV irradiation. Compared with the WT, the S93R exhibited higher RLS and ANS intensity, as well as a red-shift of E_max_, indicating that the S93R was more sensitive to UV irradiation and prone to aggregation (Figures 4D,E). The turbidities of the WT and S93R were consistent with the results of fluorescence spectra (Figure 4F). After being incubated with H_2_O_2_ for 24 h, both WT and S93R showed increase in RLS intensity, ANS intensity, and turbidity when compared to the control. However, the S93R had a relatively greater increase than the WT, indicating that the S93R mutation reduced the resistance of βB1–crystallin to oxidative stress (Figures 4G–I).

### The S93R Mutant is More Sensitive to Heat Shock, UV Irradiation, and Oxidative Stress *In Vivo*

**FIGURE 5:**
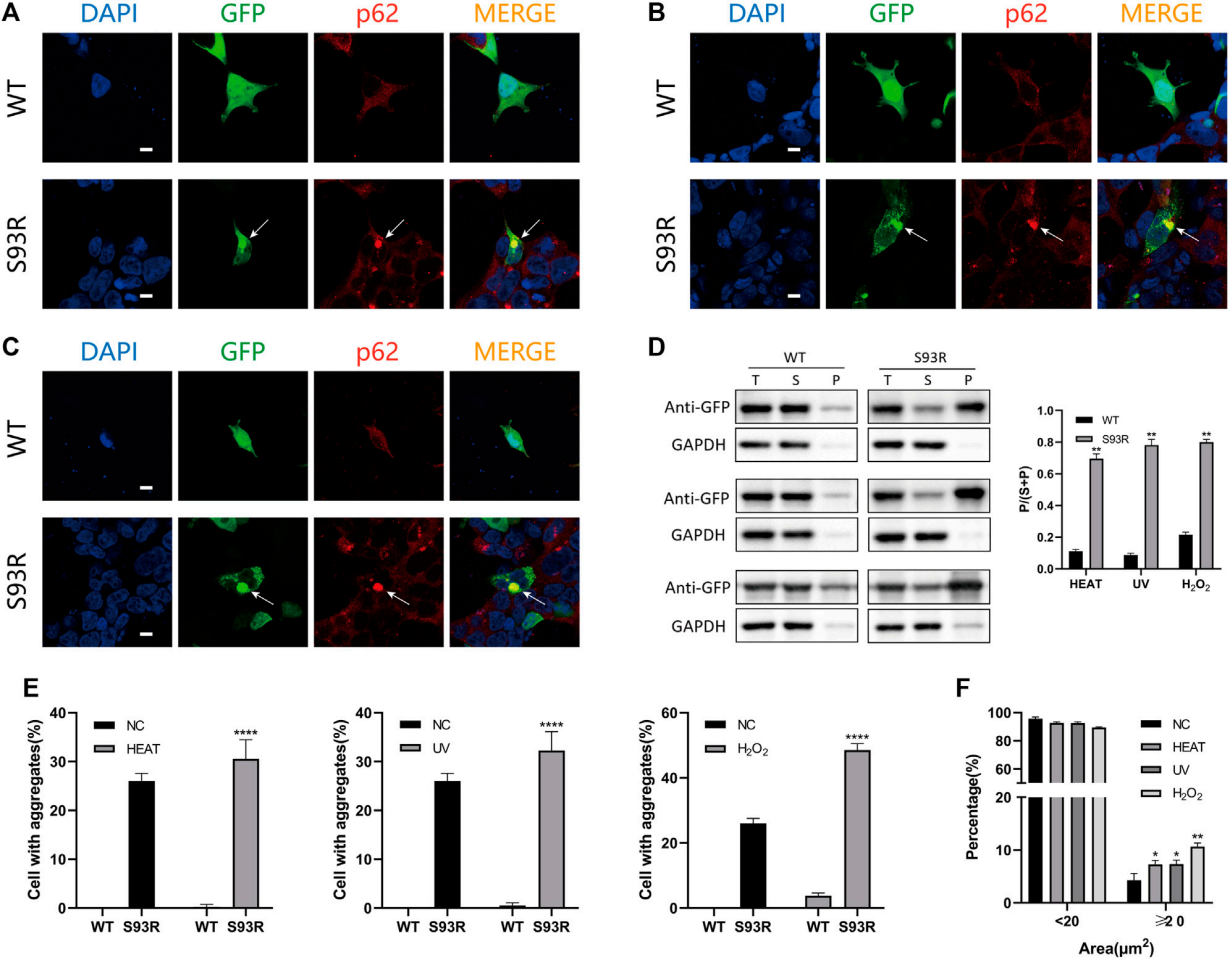
The S93R mutant was more unstable and promoted protein aggregation under heat shock, UV irradiation, and oxidative stress in HEK 293T cells. **(A)** Representative confocal images of HEK 293T cells overexpressing the EGFP-fused WT and S93R after heat shock. The cells were incubated at 42°C for 1 h. The protein aggregates are indicated by white arrows. Scale bars: 10 μm. **(B)** Representative confocal images of HEK 293T cells overexpressing the EGFP-fused WT and S93R after 30 min of UV irradiation. Scale bars: 10 μm. **(C)** Representative confocal images of HEK 293T cells overexpressing the EGFP-fused WT and S93R treated with 1 mM H_2_O_2_ at 37°C for 1 h. The medium containing H_2_O_2_ was replaced with fresh medium after treatment. Scale bars: 10 μm. **(D)** Representative western blot and densitometric results of the distribution of recombinant WT and S93R proteins overexpressed in HEK 293T cells after various treatments. Western blot results for heat shock, UV irradiation, and oxidative stress, from top to bottom respectively. T: total; S: supernatant; and P: precipitate. **(E,F)** Quantitative analysis of percentages of cells with intracellular p62-positive aggregates and the distribution of protein aggregation area after different treatments. NC: no treatment. * p < 0.05, ** p < 0.01, **** p < 0.0001.

To further mimic the occurrence of cataract, the disease cell models were subjected to various environmental stresses and visualized using immunofluorescence (Figure 5). The HEK 293T cells transfected with WT and S93R-fused plasmids were cultivated for 24 h before and 2 h after treatments to let protein aggregates form. There was no significant change in the distribution pattern of the GFP-fused WT protein after heat shock, UV irradiation, or oxidative stress, except for the appearance of some tiny protein aggregates within a few cells after H_2_O_2_ treatment. However, the S93R mutant group exhibited more intracellular p62-positive aggregates after all treatments, especially under oxidative stress (Figures 5A–C). The quantitative analysis revealed an increase in the percentages of cells with typical S93R aggregates after treatments, whereas there was no significant difference in the WT, which was consistent with the confocal results (Figure 5E). Furthermore, the distribution of protein aggregation area of the S93R suggested that the protein mutants could form larger aggregates under stressful conditions (Figure 5F). According to the western blot and the densitometric analyses, the proportion of WT proteins in the precipitate increased slightly after the treatments, but the supernatant fraction remained dominant. However, when compared to the result with no treatment, the S93R proteins showed a decrease in the supernatant fraction and an increase in the precipitate fraction. The western blot results further proved that the S93R mutant was more sensitive to heat shock, UV irradiation, and oxidative stress, all of which promoted protein aggregation in HEK 293T cells (Figure 5D).

### The Intracellular Aggregates Formed by S93R Mutant are Toxic to the Cells

**FIGURE 6:**
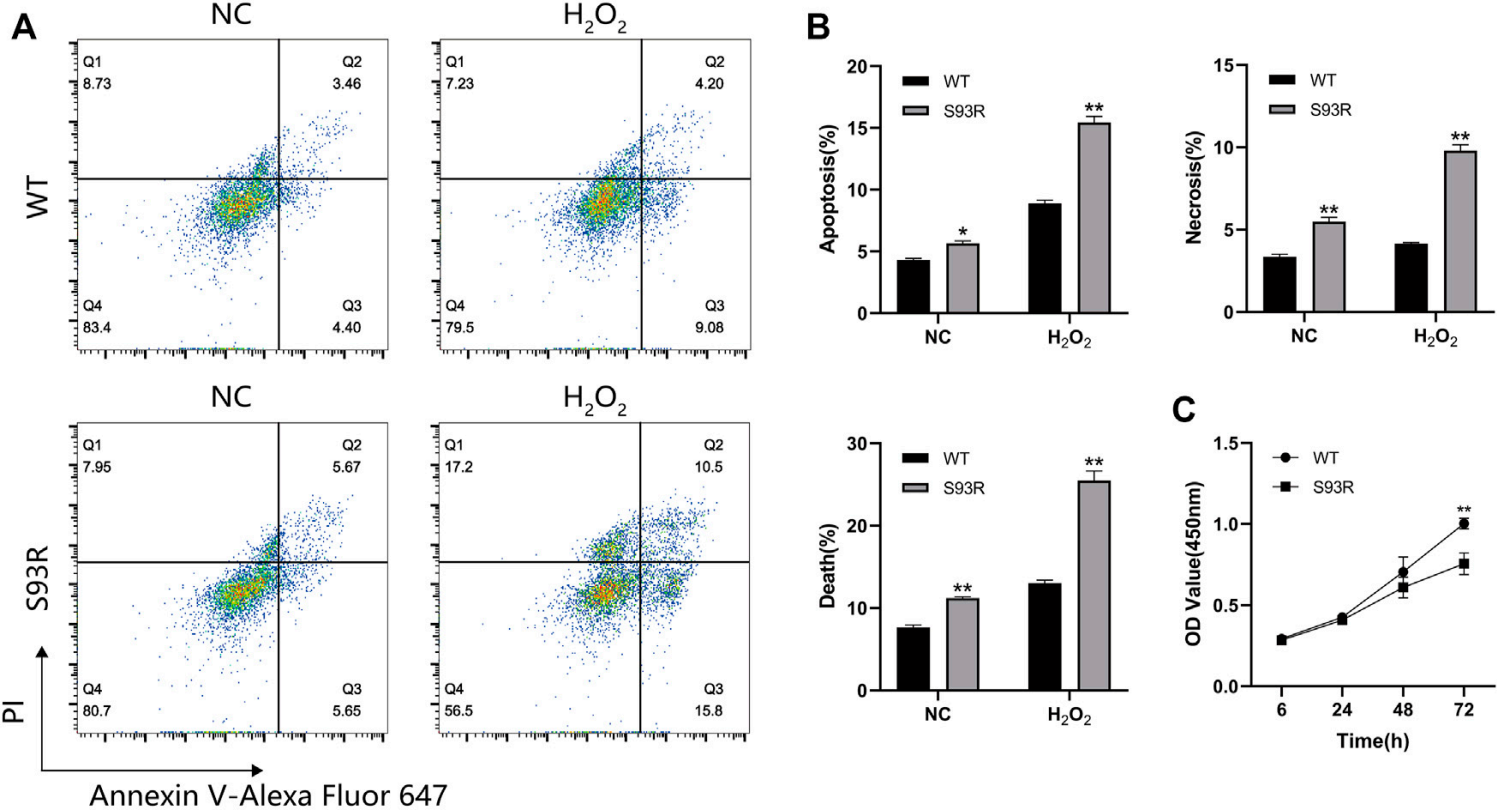
The S93R mutated proteins exogenously expressed in HEK 293T cells induced cell death and inhibited cell proliferation. **(A)** Representative flow cytometry results of HEK 293T cells transfected with GFP-tagged WT and S93R genes. The cells were double-stained with PI and Annexin V-Alexa Fluor 647. **(B)** Percentages of apoptotic, necrotic, and dead cells. The percentages of dead cells were the sum of apoptotic and necrotic cells. **(C)** Cell viability of HEK 293T cells determined by CCK-8 assay. The OD values were measured at wavelength 450 nm * means *p* < 0.05 and ** means  *p* < 0.01.

The effects of exogenously expressed S93R proteins on the survival and growth of HEK 293T cells were studied by performing flow cytometry and CCK-8 assay. Because the cells were relatively more sensitive to oxidative stress based on the confocal results and quantitative analysis, the H_2_O_2_-treated cells were also subjected to flow cytometry (Figure 6). When compared to cells expressing WT proteins, cells expressing S93R proteins had higher percentages of apoptotic and necrotic cells, as well as significantly greater increase in dead cells after H_2_O_2_ treatment, indicating that intracellular S93R aggregates induced cell death (Figures 6A,B). According to the cell viability results, the proliferation of cells expressing the S93R proteins was significantly inhibited after 72 h of cultivation (Figure 6C).

### The S93R Mutation Impaired Hydrogen Bonds Network and Structural Conformation of βB1–Crystallin

**FIGURE 7:**
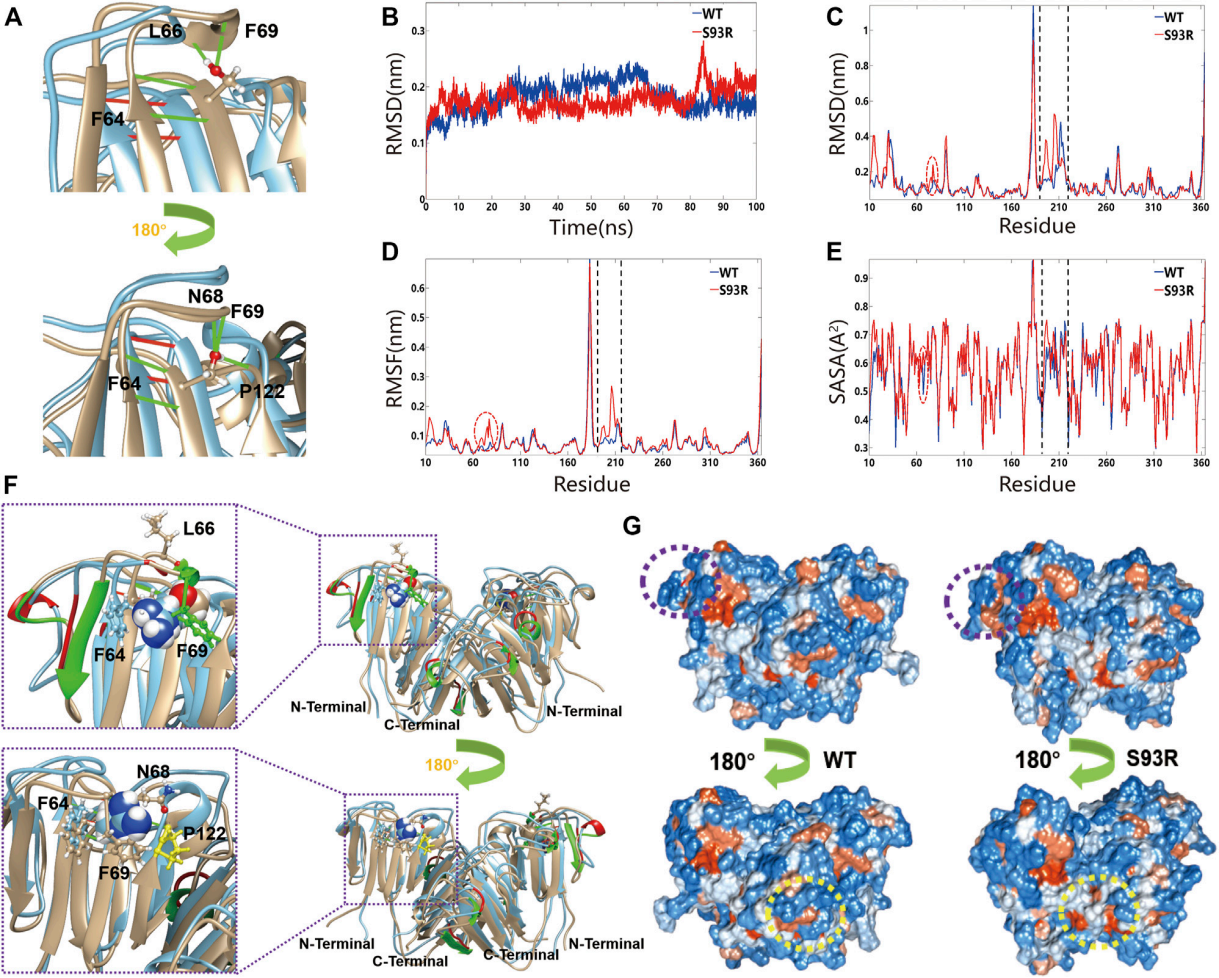
The S93R mutation caused the alteration of secondary structures and disruption of hydrogen bonds network. **(A)** The close up views of the mutation site and the affected hydrogen bonds. The residue at the mutation site is shown as balls and sticks, and the hydrogen bonds as green (formed by S93) and red (formed by R93) lines. **(B)** The time-course backbone RMSD values from the simulation structures for 100 ns. **(C–E)** The residue RMSD, RMSF and SASA of the simulated structures (residue 190 to 220). **(F)** The superposed dimeric structure of the βB1-WT (yellow) and S93R mutant (cyan). The residue at the mutation site is represented by spheres, and the residues interacting with the mutated residue through hydrogen bonds by balls and sticks. The rectangles show the close up views of the mutation site and the interacting residues. **(G)** The hydrophilic residues (blue) and the hydrophobic residues (orange) of the WT and S93R mutant at the end of the simulation. The mutation site is marked by purple dotted circles, and the dimer linkage site by yellow dotted circles.

MD simulations were applied to monitor the effects of S93R mutation on the structural stability (chain A and chain B) of βB1-crystallin. From the alignment of the dimeric structure of the WT and S93R mutant, we found that S93R mutation impaired some secondary structures of βB1-crystallin. The α-helixes (A156-F158, W174-Y176, and W215-W218) of βB1-chain A were transformed into loop structures after the S93R mutation. The α-helixes (E67-F69, S106-F108, and A156-F158) and β-strands (R73-S77, and T162-Q166) of βB1-chain B were also transformed into loop structures after the S93R mutation (Figure 7F). In addition, the S93 in the WT formed four hydrogen bonds with F64, L66 and F69 in chain B, and five hydrogen bonds with F64, N68, F69 and P122 in chain A, resulting in strong hydrogen bond networks that stabilized local β-sheet and loop structures (Figure 7A). Notably, P122 is located in the second Greek key motif of βB1-crystallin (Figures 7A,F), suggesting that the interaction between S93 and P122 might play an important role in maintaining structural stability of the N-terminal domain. However, the S93R mutant only interacted with F64 through two hydrogen bonds in both chain A and chain B (Figure 7A). Loss of the stable α-helixes, β-strands and part of the hydrogen bonds in the Greek-key motifs caused severe disruptions to local secondary structures. Furthermore, the RMSD of the S93R mutant was much higher than that of the WT from 80 ns (Figure 7B), implying that the mutation affected the protein structural flexibility. The S93R had higher RMSD, RMSF and SASA between residue 65 to 77 and 190 to 210 (Figures 7C–E). More interestingly, the S93R mutant had more hydrophobic residues at the surfaces around the mutation site and the dimer linkage site (Figure 7G). Unstable structural conformation and more exposed hydrophobic surfaces might contribute to the aggregation propensity of the S93R mutant.

## Discussion

The **β/γ**-crystallins are the main structural proteins of the lens characterized by four Greek-key motifs. The Greek-key motifs are evolutionarily conserved in vertebrates, and the structural stability and function of **β/γ**-crystallins are highly dependent on the integrity of these rigid super-secondary structures (Slingsby et al., 2013; Vendra et al., 2013; Mishra et al., 2014). Furthermore, the global structural stability of βB1–crystallin is largely dependent on acquisition of the native fold of the first Greek-key motif. Proteins fold from the N-terminus of the nascent polypeptide co-translationally. Therefore, mutations in the N-terminal may not only disrupt the local protein structure around the mutation site, but also cause misfolding of the subsequent polypeptide chain (Ingolia et al., 2019; Liutkute et al., 2020). Based on the results of molecular dynamics simulations, the stable α-helixes and β-strands were transformed into loop structures after the S93R mutation, which were much more flexible and enabled fluctuation of the local protein structures in the solvent. The alteration of secondary structures also caused more hydrophobic residue exposure at the mutation site and the dimer linkage site. In addition, disruption of the important hydrogen bonds network around the position 93 contributed to the secondary structure damage and destabilization. Overall, the S93R mutant exhibited higher flexibility and more hydrophobic residue exposure, which accounted for the high aggregation tendency of exogenously expressed S93R proteins in both human cell lines and *E. coli* Rosetta cells. In addition, completely denatured S93R proteins failed to regain their native structure during the renaturation experiment as indicated by the far-UV CD and intrinsic fluorescence spectra results. This was consistent to results from a previous protein-refolding study on βB1–S228P (Qi et al., 2016).

UV irradiation and oxidative stress are significant risk factors contributing to the occurrence of cataracts (Giblin, 2000; Lou, 2003; Chen et al., 2006; Xu et al., 2009). In previous study, the conserved W59 and W151 were proved to play important roles in β-crystallin structural integrity and stability (Zhao et al., 2017). In this work, we found that the α-helixes (W174-Y176, and W215-W218) of βB1-chain A were transformed into loop structures after the S93R mutation. Local Trp microenvironments of W174, W215, and W218 might be altered in the S93R mutant, which thereby decreased the ability of βB1-crystallin to resist UV irradiation. The increased sensitivity of the S93R to those risk factors was reinforced by additional spectral experiments exhibiting higher RLS intensity and turbidity values of the mutant after treatments of UV irradiation and H_2_O_2_, and these results were consistent with the behaviors of S93R observed within the HEK 293T cells. In addition to congenital cataracts, all affected family members developed microphthalmia in both eyes, which is a developmental anomaly of the eyes with a genetic heterogeneity (Gregory-Evans et al., 2004), (Skalicky et al., 2013). Results of the cell viability assay revealed that S93R proteins induced cell death of human cells and inhibited cell proliferation, suggesting that the S93R mutation may also influence early development of eyes.

There are seven cataract-causing mutations which have been reported in the first Greek-key motif of βB1–crystallin (Table 1) (Kumar et al., 2013; Reis et al., 2013; Lenfant et al., 2017; Javadiyan et al., 2018; Jin et al., 2020; Ji et al., 2021; Yu et al., 2021), and their underlying mechanisms might be varied. For instance, E75K affected the formation of hydrogen bonds and protein-protein interactions (Kumar et al., 2013), whereas G71S has been postulated to gain a new hydrogen bond and to lose part of the β-strand (Lenfant et al., 2017). To our knowledge, S93R was the first mutation located in the first Greek-key motif of βB1–crystallin which was purified and tested in both protein and cellular level. Currently, surgical removal of cataract lens and replacement with an intraocular lens is the only effective treatment for all types of cataracts (Chen et al., 2021) (Liu et al., 2017; Olson, 2018; Shiels and Hejtmancik, 2019). In recent years, other treatments modalities have been proposed for cataracts such as the potential ability of lanosterol to reverse crystallin aggregation (Zhao et al., 2015; Chen et al., 2018; Hu et al., 2020), and *in vivo* lens regeneration approach applied in children (Lin et al., 2016). This study provides a more comprehensive analysis of the biophysical behaviors and features of S93R mutation as well as the molecular mechanism through which it causes congenital cataracts, and can be used as a disease model for future anti-cataract drug screening.

**TABLE 1:** Summary of the reported βB1-crystallin mutations located in the first Greek-Key Motif. AD, autosomal dominant. AR, autosomal recessive.

Protein Change	Inheritance	Cataract Phenotype	Other Phenotype	Origin	Reported Year
p.V63del	Sporadic	Unknown	Microphthalmia, Pseudophakia	Sri Lanka	2018
p.Q70P	AD	Nuclear	None	China	2020, 2021
p.G71S	AR	Anterior-cortical (asymmetric)	Type 1 diabetes, glaucoma, exotropia, corneal opacity, optic atrophy	Lebanon	2017
p.E75K	Unknown	Nuclear	None	India	2013
p.D85N	Unknown	Nuclear	None	India	2013
p.S93R	AD	Unknown	Microphthalmia	China	2019
p.V96F	AD	Unknown	Glaucoma, Microcornea	United States	2013

In summary, we purified βB1–S93R proteins *via* refolding from the inclusion bodies, and analyzed the biophysical features of the mutant in comparison to wild type βB1–crystallin at both protein and cellular levels. The molecular mechanism through which the mutation contributed to the development of congenital cataracts was also explored. It was found that the S93R mutation altered the secondary structures of βB1-crystallin, increased structural flexibility and caused more hydrophobic residue exposure. The S93R mutant exhibited higher aggregation propensity, increased sensitivity to physiological temperature, UV irradiation and oxidative stress as well as potential toxicity to cells, all of which promoted the occurrence of congenital cataracts.

## Data Availability

The original contributions presented in the study are included in the article/Supplementary Materials, further inquiries can be directed to the corresponding authors.
