# Supplementary material for: Cataract-Causing S93R Mutant Destabilized Structural Conformation of βB1 Crystallin Linking With Aggregates Formation and Cellular Viability
Source: Front Mol Biosci. 2022 Mar 14;9:844719. doi: 10.3389/fmolb.2022.844719 (PMC8964140; doi:10.3389/fmolb.2022.844719)
Supplement: Supplementary file 1 [file DataSheet1.PDF]

Supplementary Material

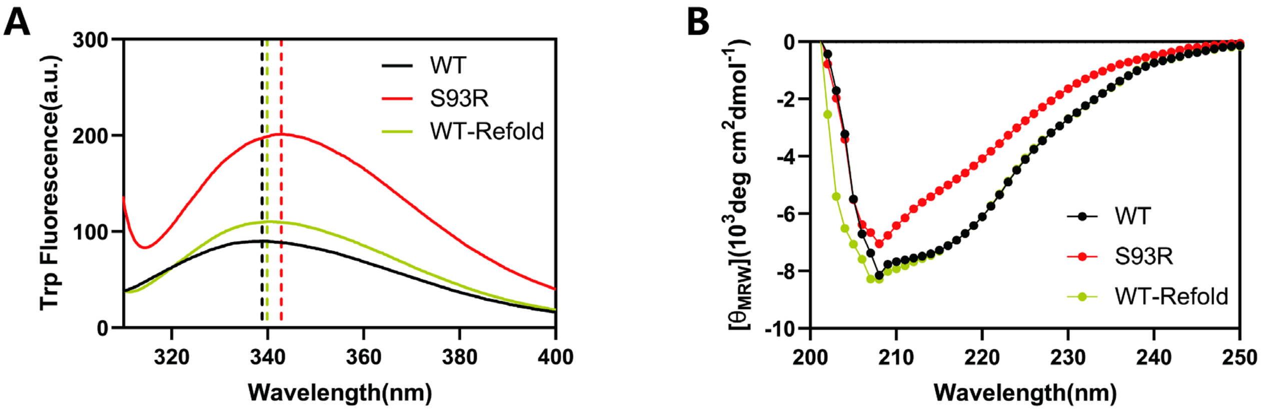

Supplementary Figure 1. Intrinsic Trp fluorescence spectra and far-UV CD spectra of the refolded WT proteins. The WT protein samples were obtained by following the same steps as the refolded S93R proteins. (A) Intrinsic Trp fluorescence spectra excited by 295 nm light. (B) Far-UV CD spectra of the refolded WT proteins. The values of CD spectra were converted into mean residue molar ellipticity ([θ_{MRW}]).

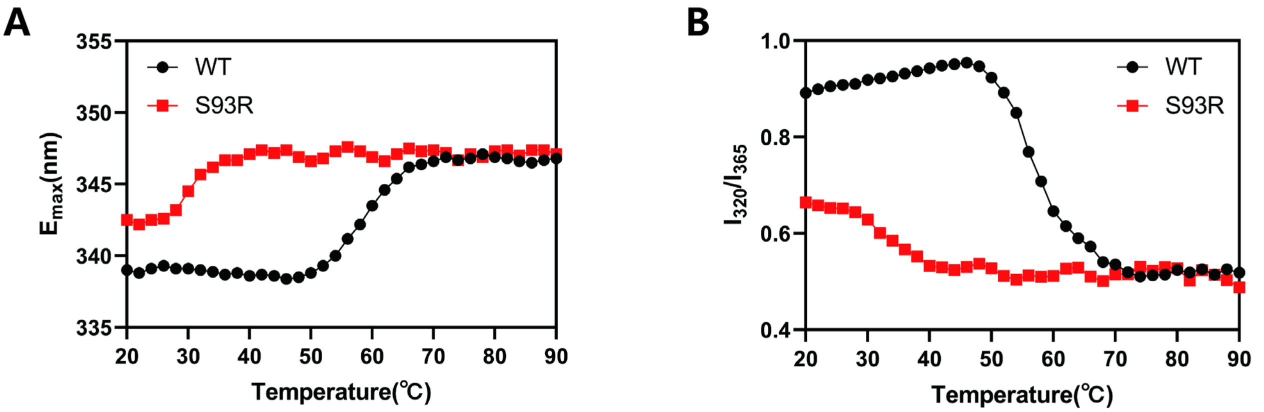

Supplementary Figure 2. The temperature-gradient heating experiments. The protein samples were heated stepwise from 20°C to 90°C at an interval of 2°C and incubated for 2 min at each temperature before measurement. (A) E_{max} curves of the WT and S93R Trp fluorescence. (B) I_{320}/I_{365} curves of the WT and S93R Trp fluorescence. I_{320}/I_{365}： the ratio of Trp fluorescence intensity at 320 nm wavelength with that at 365 nm wavelength.
